# Sustained Attention in Real Classroom Settings: An EEG Study

**DOI:** 10.3389/fnhum.2017.00388

**Published:** 2017-07-31

**Authors:** Li-Wei Ko, Oleksii Komarov, W. David Hairston, Tzyy-Ping Jung, Chin-Teng Lin

**Affiliations:** ^1^Institute of Bioinformatics and Systems Biology, National Chiao Tung University Hsinchu, Taiwan; ^2^Institute of Molecular Medicine and Bioengineeing, National Chiao Tung University Hsinchu, Taiwan; ^3^Brain Research Center, National Chiao Tung University Hsinchu, Taiwan; ^4^Swartz Center for Computational Neuroscience, University of California, San Diego, San Diego CA, United States; ^5^Human Research and Engineering Directorate, Army Research Lab, Aberdeen WA, United States; ^6^Centre for Artificial Intelligence, Faculty of Engineering and Information Technology, University of Technology Sydney, Sydney NSW, Australia

**Keywords:** EEG, classroom, real environment, sustained visual attention, mental fatigue, response time, spectral analysis

## Abstract

Sustained attention is a process that enables the maintenance of response persistence and continuous effort over extended periods of time. Performing attention-related tasks in real life involves the need to ignore a variety of distractions and inhibit attention shifts to irrelevant activities. This study investigates electroencephalography (EEG) spectral changes during a sustained attention task within a real classroom environment. Eighteen healthy students were instructed to recognize as fast as possible special visual targets that were displayed during regular university lectures. Sorting their EEG spectra with respect to response times, which indicated the level of visual alertness to randomly introduced visual stimuli, revealed significant changes in the brain oscillation patterns. The results of power-frequency analysis demonstrated a relationship between variations in the EEG spectral dynamics and impaired performance in the sustained attention task. Across subjects and sessions, prolongation of the response time was preceded by an increase in the delta and theta EEG powers over the occipital region, and decrease in the beta power over the occipital and temporal regions. Meanwhile, implementation of the complex attention task paradigm into a real-world classroom setting makes it possible to investigate specific mutual links between brain activities and factors that cause impaired behavioral performance, such as development and manifestation of classroom mental fatigue. The findings of the study set a basis for developing a system capable of estimating the level of visual attention during real classroom activities by monitoring changes in the EEG spectra.

## Introduction

Human behavior and adaptability to varying environmental conditions require complex cognitive activities that occur in both highly developed neocortex and phylogenically older structures of the brain. The ability to focus on specific stimuli in order to complete a planned task is an example of such complex cognitive activity, which is called sustained attention. Sarter et al. (2001) defined sustained attention as a fundamental component of attention characterized by readiness to detect rarely and unpredictably occurring signals over prolonged periods of time.

Processing of visual information is a multistage physiological process, which involves multiple brain areas. It has been shown in the monkey that the striate cortex is a source of two multisynaptic cortical pathways or streams (Ungerleider and Mishkin, 1982; Mishkin et al., 1983). The faster one courses dorsally and processes the location of objects. The slower one runs ventrally and is responsible for visual identification of the observable objects. Subsequent studies proved this hypothesis in humans. The dorsal stream was described as coursing through the occipito-parietal cortex to the posterior part of the inferior parietal lobule, with a likely further extension to the dorsolateral prefrontal cortex. The ventral stream was described as coursing through the occipito-temporal cortex to the anterior part of the inferior temporal gyrus, with a likely extension to the ventrolateral prefrontal cortex (Macko et al., 1982; Goodale et al., 1994). According to general functional characterization of the dorsal stream, which is associated with visually guided behavior, it has been defined as the “where” pathway; the ventral stream has been defined as the “what” pathway with a function of supporting the processing of object quality or identity (de Haan and Cowey, 2011; Kravitz et al., 2011). One of the most consistent findings regarding the intrinsic function of the occipito-temporal network (Tsao et al., 2008; Bell et al., 2011) is the presence of functional clustering in the cortex with selectivity for particular object categories, such as body parts and faces, scenes, geometric objects, tools, written words, colors (Malach et al., 1995; Epstein and Kanwisher, 1998; Downing et al., 2006; Conway et al., 2007; Mahon et al., 2007; Murphey et al., 2008; Dehaene and Cohen, 2011). It is known that visual attention, whether directed to spatial locations (Gandhi et al., 1999), objects (Shomstein and Behrmann, 2006), or other unique features (Maunsell and Treue, 2006), affects the visual information processing directly within corresponding areas of the occipito-temporal network involving the mechanisms of memory (Kravitz et al., 2013). An electrophysiological study of the visual pathway mechanisms revealed the reverse order of attentional effects, such that attentional enhancement of neural firing rates was larger and earlier in V4 and smaller and later in V1, with intermediate results in V2, which suggests that attentional mechanisms operate via feedback from higher-order areas to lower-order ones (Buffalo et al., 2010).

Conducting experiments under laboratory conditions for measuring the brain activities is a widely used approach in cognitive neuroscience studies. However, many efficient research techniques, such as PET or fMRI, are not suitable if one wants to investigate brain activation patterns as they relate to real-world environments. To make visualization of the brain dynamics during natural social situations feasible and reliable, it is required to apply special sophisticated real-world neuroimaging techniques (McDowell et al., 2013; Kasai et al., 2015). One of the most common electrophysiological methods to study the brain activity is an identification the spectral characteristics of the electroencephalographic (EEG) signals and the brain regions where there is a change in these characteristics under different conditions. It has been shown that activities in different EEG frequency bands can be related to specific physiological states. The EEG oscillations have been classified by their frequencies since the very early studies after the discovery of EEG (Adrian and Mathews, 1934). The technological development of recent digital signal processing methods resulted in a more precise quantification of EEG frequencies and the definition of frequency bands that have the approximate following ranges: 1–3 Hz for delta, 4–7 Hz for theta, 8–14 Hz for alpha, 15–30 Hz for beta and higher than 30 Hz for gamma. The oscillatory electrocortical activity in all frequency bands is considered to be linked to a broad variety of perceptual, sensorimotor, and cognitive operations, including attention and memory (Aoki et al., 1999; Klimesch, 1999; Osipova et al., 2006; Palva and Palva, 2007).

As any living creatures, people cannot maintain their optimal attention for a long time without falling into a state of fatigue (Mackworth, 1948). There are different ways to classify fatigue – it can be short-term or long-term, physical or mental (Jason et al., 2010). As opposed to physical fatigue, which is usually associated with the whole body or muscles stamina exhaustion, mental fatigue is the one associated with a reduced efficiency of “brain work” – intensive brain activity focused on the execution of a specific task or tasks. Fatigue itself is rather an alarm signal of the necessity of a rest; however, its prolonged presence may lead to undesired consequences. With the rapid development of the modern world, people have to process large amounts of information, so that they fall into a risk group of experiencing negative effects of mental fatigue. According to community-based epidemiological studies, from 15 to 25% of the general population report that they feel mental tiredness, which can be classified as short-term fatigue; 5–18% and 3–10% of the general population experience prolonged and chronic (longer than 1 months) fatigue respectively (Fukuda et al., 1997; Kant et al., 2003). A well-known fact is that it is particularly easy to get into a state of mental fatigue during execution of work that requires maintaining a high level of concentration for a long time, such as studying, driving, writing or reviewing of scientific papers. Mental fatigue may have various physiological causes and signatures, including both impaired electrocortical activity in task-related brain regions (Tanaka et al., 2015) and extensive changes in the EEG oscillations (Klimesch, 1999). It has been reported that attention is affected by mental fatigue in the form of a decrease in the ability to suppress irrelevant information and inhibit shifts of attention to irrelevant stimuli, which leads to an increase in reaction times and the number of incorrect responses (Boksem et al., 2005; Faber et al., 2012). The problem of fatigue-related studies is compounded by the fact that the reaction to fatigue is highly variable across individuals and depends on multiple factors, such as motivation in goal-directed tasks. In our previous studies, we demonstrated the relationship between impaired visual attention and the effect of task-induced mental fatigue (Pal et al., 2008; Lin et al., 2012), which led to lapses in a driving task. It was shown that changes in the theta and low alpha spectral power in the occipital and parietal regions accompanied the mental fatigue and resulted in declines of the visual attention task performance.

Meanwhile, in the modern world, education is considered as an essential stage in the development of personality. The processes of learning and memorization are closely related to the ability to concentrate selectively on a discrete aspect of information, while ignoring other perceivable information. The use of projectors in classrooms – particularly at the upper grades and collegiate level – to display lecture materials became the generally accepted standard over the last decade. Such an approach allows teachers to introduce new information in a more efficient way, but at the same time may increase students’ mental strain requiring them to focus on the screen during the whole lecture. Paying attention to these lectures is functionally a sustained-attention task of its own. However, the presence of a variety of distraction factors in a real classroom makes it a challenge to examine the brain activity changes related to variations in attention level and verify the conclusions obtained in the laboratory. A recent study of group interactions in the classroom demonstrated a possibility to predict student class engagement by assessing group-based neural coherence and considering brain-to-brain synchrony as a possible neural marker for dynamic social interactions (Dikker et al., 2017).

In order to extend the aforementioned work (Lin et al., 2010) for developing a generalized human attention model and to investigate electroencephalographic correlates of sustained attention in real-world situations, we set a real classroom as the experimental environment in this study. This study systematically explores tonic spectral variations, which slowly occur over time in the absence of any particular discrete environmental event or external stimuli (Rajkowski et al., 1994; Makeig and Jung, 1996), in the delta, theta, alpha, and beta frequency bands to assess specific links between cortical brain activities (via changes in the EEG spectral power) and performance (reaction time) in a visual attention task. The results may provide insights into the development of brain–computer interfaces (BCI) for assessing students’ level of alertness and visual attention (Jung et al., 1997; Lin et al., 2006).

## Materials and Methods

### Subjects

Eighteen healthy volunteer participants (11 males and 7 females) aged from 23 to 27 years took part in this study. All the participants were right-handed, had normal or corrected to normal vision and no history of neurological or psychiatric disorders. The experiment was performed in accordance with the country’s laws and approved by the institutional review board (IRB) of the National Chiao Tung University (NCTU). Each participant provided written informed consent prior to participation. The participants were compensated approximately 20 USD after the experiments.

### Classroom Activities

To implement the use of sustained attention for classroom activities in the most realistic way, the experiments were carried out during regular lectures held at NCTU. All the participants were students taking the course. Evaluation of academic performance on the course was performed without distinctions between participants and non-participants of the experiment. The participants of this study were naturally motivated to give their full attention to the perception of lecture materials in order to achieve good scores on exams and successfully pass the course. All data collection was conducted during one academic semester. Experimental results were not used for evaluating the participants’ study performance and were kept confidential and anonymous to avoid any effect on the scoring of the coursework.

### Visual Attention Task

**FIGURE 1:**
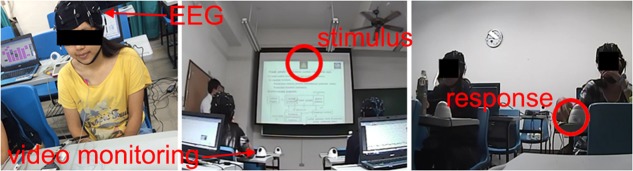
Performance of the visual attention task in a real classroom environment. Video monitoring and EEG recordings were conducted during experimental sessions. The participants were instructed to press the corresponding buttons as quickly as possible after the visual targets appearances.

**FIGURE 2:**
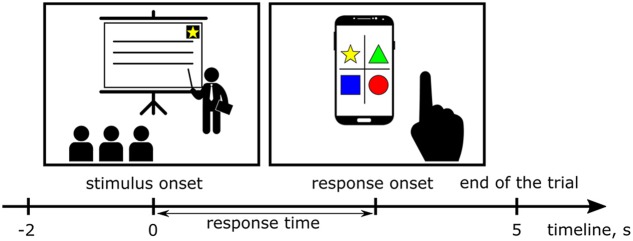
During the lectures, images of simple geometric objects (stimuli) appeared on the screen. Each participant had a smartphone with an application that displayed all possible choices in the form of buttons with pictures. The time interval between an appearance of the stimulus on the screen and pressing the button defined the response time (RT). Two second long intervals of the EEG records immediately preceding appearances of the stimuli were extracted as epochs to investigate tonic changes in the EEG power spectra.

To assess the level of visual alertness, we used a specially designed test for visual targets recognition. During the lectures, images of simple geometric objects (stimuli) appeared on the screen with random time intervals, but no more often than one stimulus per minute (average inter-trial interval 92 ± 33 s, 36 ± 10 trials per session). There were four types of the stimuli (triangle, square, circle and star) and only one of them was shown in a single trial (**Figure 1**). Each participant of the experiment had a smartphone with an application that displayed all possible choices in the form of buttons with pictures and kept the smartphone awake and unlocked during the whole experimental session. Before each experimental session, the participants were instructed to stay alert and press the corresponding buttons as quick as possible if they notice the stimuli, but they were neither rewarded nor punished for good or bad performance in the test to make sure that it did not affect their primary task of perceiving the lecture materials. The time interval between appearance of the stimulus on the screen and pressing the button defined the response time (RT, **Figure 2**). Longer RT indicates lower level of visual alertness and presumably higher level of drowsiness (Sarter et al., 2001). The trials, in which the response was not received within five seconds after the presentation of the stimulus or the response did not match the stimulus, were classified as missed and excluded from further analysis. All devices were connected to the same network and synchronized for coordinated stimuli presentation, response collection and EEG recording. Timestamps of the stimuli appearances and responses of the participants were automatically tagged as event markers in their respective EEG records. Video recordings of the participants’ faces were manually reviewed in order to ensure that long RTs were indeed related to impaired reaction time, but not from distraction or other factors. The duration of an experimental session was 50 minutes (a typical college class at NCTU). Due to pragmatic limitations, the number of sessions collected was not the same for all participants, ranging between 2 and 8 (mean 5.4), with the majority performing 6. One of the subjects took part in only one experimental session; the corresponding dataset was not used in the data analysis. **Table 1** summarizes the number of sessions by subjects and average response times in trials with correct responses.

**Table 1:** Summary of sessions collected from each subject.

Subject ID	Mean alert state RT, ms	Mean ± STD RT, ms	Number of sessions
1	1441	2161 ± 570	6
2	1229	2061 ± 598	5
3	1581	2172 ± 439	8
4	1155	2447 ± 674	7
5	1597	2404 ± 630	7
6	961	1963 ± 776	6
7	1727	2278 ± 477	5
8	1506	2175 ± 486	6
9	1768	2337 ± 446	6
10	1829	2395 ± 419	4
11	1498	2228 ± 589	6
12	1884	2576 ± 571	6
13	1931	2576 ± 581	6
14	1185	1994 ± 538	3
15	1347	2065 ± 672	2
16	944	1523 ± 319	5
17	1218	1885 ± 608	4

Total	1459 ± 302	2188 ± 624	92

*In the **total** row, the mean alert state RT averages mean alert state RTs of each subject, while the mean RT is calculated using the whole dataset*.

### EEG Collection

The EEG signals were recorded using Ag/AgCl electrodes attached to a 32-channel Quik-Cap (Compumedical NeuroScan). Thirty EEG electrodes were placed according to the extended International 10–20 System with two reference electrodes located on the both mastoid bones. The skin under the reference electrodes was abraded using Nuprep (Weaver and Co., United States) and disinfected with a 70% isopropyl alcohol swab before calibration. The impedance between electrodes and skin was kept below 5 kΩ using NaCl-based conductive gel (Quik-Gel, Neuromedical Supplies^®^). EEG signals from the caps were amplified using a Scan NuAmps system (Compumedics Ltd., VIC, Australia) and recorded at a sampling rate of 1,000 Hz. The data processing was performed using EEGLAB (Delorme and Makeig, 2004), a toolbox for MATLAB. The collected EEG data were inspected for artifacts and noise-corrupted channel signals, subjected to 1 Hz high-pass and 50 Hz low-pass basic FIR filters to remove muscular artifacts and power grid noise, then down-sampled to 250 Hz. After quality inspection of the corresponding EEG datasets, 92 experimental sessions from 17 subjects were selected for further analysis. To calculate the parameters of tonic power spectral changes in the brain activity, we extracted 2 s long intervals of the EEG records immediately preceding the appearance of the stimuli as epochs. Each EEG sample was converted to the frequency domain by Fast Fourier Transform (FFT) in the range from 1 to 30 Hz with a step of 1 Hz to investigate its spectral characteristics.

### Statistical Analysis

Averaged over the EEG frequency bands spectral powers were compared using a one-way ANOVA with *post hoc t*-tests. The comparable distributions passed the Lilliefors test for normality (Lilliefors, 1967). All significance tests were corrected for multiple comparisons using the false discovery rate (Benjamini and Hochberg, 1995). The alpha level of 0.05 was used for statistical hypothesis testing in this study unless otherwise specified.

## Results

### Response Time

**FIGURE 3:**
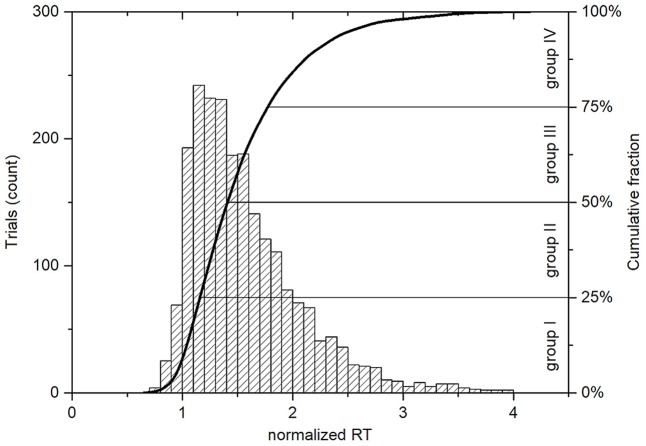
Histogram and cumulative distribution plot of normalized response times for all trials with correct responses (*n* = 2212). The normalized RT is unitless and is calculated for each trial. It represents how many times the RT is greater than the corresponding mean alert state RT, which is individual for each subject. The Q_1_ = 1.21, Q_2_ = 1.45, and Q_3_ = 1.83 values set the boundaries between the four groups, where the group I corresponds to the highest level of visual alertness and the group IV contains trials with the longest normalized RTs.

Before the experiment, all subjects were instructed to stay as alert as possible and respond to the visual stimuli as quickly as possible. Since the response times were not normally distributed and varied widely across individuals (Heathcote et al., 1991), we used the concept of the alert state (Pal et al., 2008; Lin et al., 2013) in order to perform group analysis of the data. During an experimental session, subjects had to respond to multiple visual attention events and still pay attention to lecture materials, so their performance tended to fluctuate over time. Ten percent of trials with the shortest RTs of each participant were considered as baselines or the states of maximal alertness, in which the subjects were fully attentive to the given task. All RTs from these baselines were averaged as the mean alert RTs for each individual (**Table 1**). To sort the experimental trials according to the level of visual alertness taking into account variations in individual reaction abilities of each participant, the response times of all sessions from each individual were normalized by a division by the mean alert state RTs. After that, all trials were distributed into four equal of size groups, separated by three quartiles: Q_1_, Q_2_ and Q_3_. The first 25% of all trials (normalized RTs < Q_1_) were allocated to the group I that corresponded to the highest level of visual alertness. 25% of the trials with normalized RTs > Q_3_ formed the group IV representing the worst performance in the classroom visual attention task. The trials with normalized RTs between Q_1_ and Q_3_ values were distributed to the groups II and III, being separated by the median or Q_2_, which corresponded to 1.45 times the mean alert state RT (**Figure 3**).

### Tonic Changes in EEG Power Spectra

**FIGURE 4:**
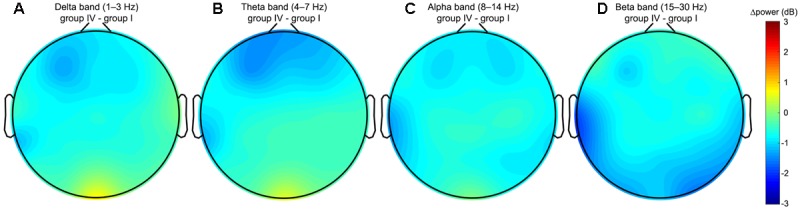
Topoplots of the difference in tonic powers between the groups IV and I. The topoplots are built using the whole set of EEG channels and represent the spectral power variations in the **(A**) delta, **(B)** theta, **(C)** alpha, and **(D)** beta frequency bands. The comparison of the two groups reveals an increase in the delta and theta powers in the occipital region, decrease in the delta, theta and alpha powers in the frontal region, decrease in the beta power in the temporal and occipital regions associated with an increase in the normalized RT in the visual attention task.

**FIGURE 5:**
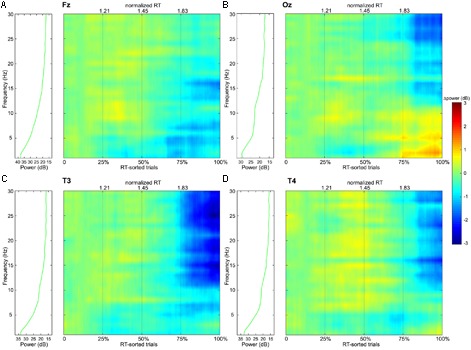
Frequency-power colormaps for the EEG power spectra at single channels (**A**: Fz, **B**: Oz, **C**: T3, and **D**: T4) that are located in the frontal, occipital, left and right temporal regions respectively. The colormaps represent matrixes, where the columns correspond to experimental trials sorted according to their normalized RTs from left to right, and the rows correspond to frequencies from 1 to 30 Hz with a step of 1 Hz. The plots are separated into four independent windows by the thin gray vertical lines that represent the Q_1_, median and Q_3_ values of the normalized RT distribution (**Figure 3**). The green curves on the left panels show average spectral power distributions in the 1–30 Hz range for the group I, which are considered as the baselines – the colormaps themselves show power deviations from the first group’s averages.

**FIGURE 6:**
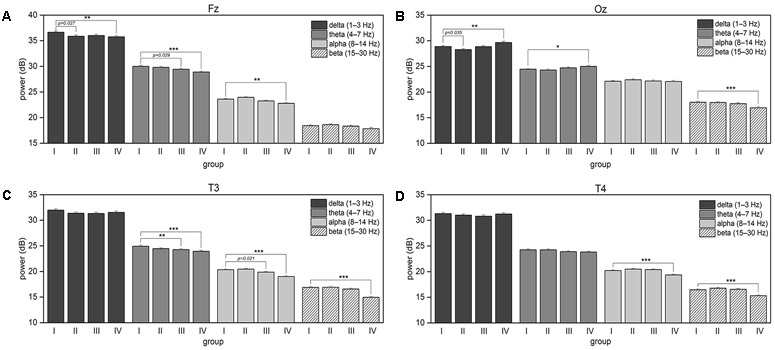
The plots show associated with an increase in the response time changes in the four frequency bands’ mean power spectra for **(A)** Fz, **(B)** Oz, **(C)** T3, and **(D)** T4 EEG channels. The error bars represent the standard error of the mean. The asterisk brackets indicate significance according to *post hoc t*-tests that compare each group with the group I (first 25% of sorted by normalized RT trials) within the same EEG frequency band; the *p*-values below 0.05 are shown for the tests rejected after false discovery rate correction.

The continuous EEG signals were segmented into 2-s trials, from 2 s preceding and to the events of the visual attention targets appearance. We specifically use the data preceding the event, and not following the event, in order to investigate the neural state of alertness prior to stimulation, without any contamination from the perceptual or motor response. The EEG signals that were considerably contaminated by artifacts (such as muscle activity, blinks, eyes movement, and environmental noise) were manually eliminated to minimize their influence on subsequent analysis. To reveal changes in the tonic brain activity depending on the level of alertness, all obtained EEG samples were distributed into four independent equal size groups according to the procedure described in the Section “Response Time”. Time courses of EEG channels activations were then transferred to the frequency domain using the FFT. The resultant time-frequency estimates consisted of 30 frequency bins from 1 to 30 Hz with a step of 1 Hz covering the delta (1–3 Hz), theta (4–7 Hz), alpha (8–14 Hz), and beta (15–30 Hz) EEG frequency bands. **Table 2** summarizes the results of post-hoc *t*-tests for the certain brain areas, where significant differences between tonic EEG powers associated with long and short response times in the visual attention task were detected according to a one-way ANOVA. As the reaction time increases, tonic power spectra registered with EEG channels from the occipital, temporal and frontal regions vary. **Figure 4** shows the differences in tonic power spectra between the groups IV and I for the four EEG frequency bands in the form of topographic maps. The observed tonic power changes in each frequency band in comparison with the mean power spectra of the first 25% epochs (group I) are discussed below (**Figures 5, 6**).

**Table 2:** Summary of *post hoc t*-tests for the groups IV and I.

	δ	θ	α	β
Frontal	-	-	-	
Left temporal		-	-	-
Right temporal			-	-
Occipital	+	+		-


*Symbols – and + indicate that the mean spectral power in the group IV is significantly **less** or **greater** than that in the group I respectively*.

#### Delta Band Power (1–3 Hz)

The delta power decreases in the frontal region with an increase in the normalized RT. In the occipital region, the delta power fluctuates, dropping down in the second quarter of trials but then starts to increase as the RTs increase.

#### Theta Band Power (4–7 Hz)

The theta power monotonically decreases in the frontal and left temporal regions. An increase in the theta power is observed in the occipital region comparing the groups IV and I.

#### Alpha Band Power (8–14 Hz)

Mean spectral power in the alpha band fluctuates in the frontal and temporal regions. Mean alpha band power remains unchanged as the RT increases moderately, and decreases when the RTs are long.

#### Beta Band Power (15–30 Hz)

As the response time in the visual attention task increases, mean baseline power in the beta band decreases significantly in the last quarter of trials over the occipital and both temporal brain regions compare to the first quarter of trials.

**FIGURE 7:**
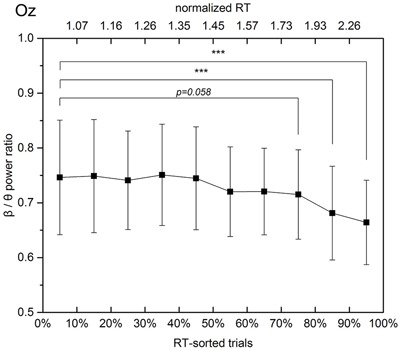
Beta to theta band power ratio at the Oz channel in experimental trials sorted according to the respective normalized RTs. The solid line linearly interpolates 10 mean values, which are calculated with a step of 10% of trials. The error bars show the standard deviations in the subgroups separated by the deciles. When the response time exceeds 1.3 times the mean alert state response time, the mean beta to theta power ratio decreases monotonically and steadily from 0.75 (30–40% subgroup, average normalized RT equals 1.3) to 0.66 (90–100% subgroup, average normalized RT equals 2.7).

Since both the decrease and increase in spectral powers were observed in the occipital area, **Figure 7** demonstrates changes in the β/𝜃 power ratio that accompany the RT elongation.

## Discussion

In the previous work, we investigated fatigue-related tonic changes in the EEG power spectra in a virtual-reality-based highway-driving environment (Pal et al., 2008; Lin et al., 2013). The results showed that tonic power increased in the delta, theta, and alpha bands in the occipital and posterior parietal cortices when reaction times in the visual attention task increased. In this classroom study, no spectral variations were found in the posterior parietal area, which is known to play an important role in visual orientation and spatially oriented behavior (Constantinidis et al., 2013). This discrepancy may be explained by different natures of the implemented tasks: classroom activities involve complex cognitive mechanisms including recognition, processing and memorization of new information, while simulated driving requires from subjects to maintain sustained spatial attention and judge the car’s position on the road.

In a laboratory study of attention and attention deficits, Gola et al. (2013) revealed that the beta band power of EEG signals, recorded over the occipital region, was related to visual attention, as judged from an increase in the beta power preceding correct responses and lack of beta activity changes before erroneous responses. According to Bekisz and Wróbel (1993), beta activity is related to attention and attention deficits not only in humans or monkeys, but also in cats: the beta-band power increased during the anticipatory time period of a visual spatial differentiation task in trials resulting in correct responses. It was shown that beta band activity may serve as a carrier for attentional activation in many thalamic and cortical centers of the visual system (Bekisz and Wróbel, 1999, 2003; Wróbel, 2000). According to a PET study of functional interactions within cortical networks, which are responsible for visual perception, dominant path influences were focused among occipito-temporal areas in the object vision task (McIntosh et al., 1994). The results of this classroom experiment show significant beta activity changes over the temporal and occipital lobes, where increased beta band power correlated with a higher level of visual attention. Carrying out such an experiment demonstrates the possibility of observing visual attention-related changes in the brain activities using the method of EEG in an everyday scenario.

The brain lateralization plays an important role in a majority of complex cognitive functions (Ocklenburg and Güntürkün, 2012). It has been shown that the hemispheric asymmetry is relevant to language processing (Vikingstad et al., 2000), working memory (Habib et al., 2003), motor skills (Crone et al., 1998), and visually guided behavior (Asanowicz et al., 2013). Bethmann et al. (2007) determined that about 95% of right-handers exhibited left hemispheric language dominance. The results of this study reveal more intensive EEG spectral power variations in a wider frequency range in the left hemisphere, in particular over the temporal lobe. While an increase in the response time in the visual attention task was accompanied by a significant decrease in the average alpha and beta powers in the both temporal regions, no significant differences in the theta band power were observed over the right temporal lobe (*p* = 0.070) despite a trend for decreasing. Taking into account the complexity of cognitive load in the classroom, it is possible to make an assumption about the presence of laterally asymmetric cortical activities related to the process of studying during lectures.

In a study of neural responses evoked by multiple presentations of short film clips to a group of subjects, Dmochowski et al. (2012) formulated a novel signal decom position method based on an extraction of maximally correlated signal components from multiple EEG records. Recently the method of inter-subject correlation was implemented in the classroom to demonstrate that stimulus-evoked neural responses, which are known to be modulated by attention, could be tracked for groups of students using synchronized EEG acquisition (Poulsen et al., 2017). A limitation of this study is that it dealt with a rather artificial task of movie clips presentation, which did not represent real classroom activities. However, it proved the possibility of using portable wireless consumer grade EEG equipment to assess attention-related brain activities in a real environment, as it was shown in previous studies (Yasui, 2009; Debener et al., 2012).

Electroencephalography-based BCI systems use acquisition and analysis of electrical brain activity to create a new direct output channel from the brain to a computer. While active BCIs require intentional execution of a special task for controlling the connected computer system or communication through it, passive or implicit BCIs perform cognitive monitoring utilizing real-time brain signal decoding for gaining information about the ongoing user state (Zander and Kothe, 2011). The simplest of them are based on the estimation of the EEG spectral power in certain frequency bands and have a wide range of implementations (Pal et al., 2008; McDowell et al., 2013). For example, systems that use such principle have been proven to be effective in determining the level of a driver’s drowsiness (Lin et al., 2013) and predicting the declined performance in a vehicle operation (Saproo et al., 2016). The obtained results show the presence of easily extractable features, which change monotonically as behavioral performance declines (such as beta to theta power ratio over the occipital region, **Figure 7**). Such features can be considered as markers and the presence of a significant difference between them in different cognitive states, such as attention or inattention, allows them to be used for training a machine-learning algorithm. As the next step, it can be applied to solve the inverse problem and estimate the reaction time based on the EEG signal. This opens the door for development a system that is capable to estimate the level of visual alertness in an everyday classroom scenario.

## Conclusion

The results of this study revealed significant tonic spectral changes in trials with long response time compared to the trials, in which the participants were in the alert state. Comparing the obtained results with results of other visual attention studies it is possible to determine both similar and dissimilar patterns of the brain activations, which allows us to make an assumption about the role of the ventral pathway of the visual information processing in the described cognitive task. It has been shown that a decrease in the resting state beta band activity in the task-related brain regions precede the declined behavioral performance. The study demonstrates feasibly of performing the described sustained visual attention task in a highly distractive real classroom environment. The next suggested phase of the study is to examine the influence of the mental fatigue as it changes over time on behavioral performance and associated cognitive activity. The obtained results can be applied for developing a BCI system capable of estimating the level of visual attention and related drowsiness in a real classroom environment by monitoring changes in the EEG spectra.

## Ethics Statement

This study was carried out in accordance with the recommendations of institutional review board (IRB) of National Chiao Tung University, Hsinchu, Taiwan. All subjects gave written informed consent in accordance with the country’s laws. The experimental protocol was approved by the IRB and assigned the number NCTU-REC-103-025.

## Author Contributions

All authors were contributing to the drafting and revising of the documents and approved the final version. Everybody agreed to be accountable for the integrity and accuracy of the work. L-WK and C-TL proposed the initial idea and designed the experiment for studying the generalized human attention and fatigue model. L-WK and OK performed the data collection, analysis and wrote the manuscript. WDH and T-PJ are consultant for quality of writing and validating the results.

## Conflict of Interest Statement

The authors declare that the research was conducted in the absence of any commercial or financial relationships that could be construed as a potential conflict of interest.

## References

[B1] Adrian E. D. Mathews B. H. C. (1934). The interpretation of potential waves in the cortex. *J. Physiol.* 81 440–471. 10.1113/jphysiol.1934.sp003147 16994555 PMC1394145

[B2] Aoki F. Fetz E. E. Shupe L. Lettich E. Ojemann G. A. (1999). Increased gamma-range activity in human sensorimotor cortex during performance of visuomotor tasks. *Clin. Neurophysiol.* 110 524–537. 10.1016/S1388-2457(98)00064-9 10363776

[B3] Asanowicz D. Smigasiewicz K. Verleger R. (2013). Differences between visual hemifields in identifying rapidly presented target stimuli: letters and digits, faces, and shapes. *Front. Psychol.* 4:452 10.3389/fpsyg.2013.00452 PMC3715728 23882249

[B4] Bekisz M. Wróbel A. (1993). 20 Hz rhythm of activity in visual system of perceiving cat. *Acta Neurobiol. Exp.* 53 175–182. 8317245

[B5] Bekisz M. Wróbel A. (1999). Coupling of beta and gamma activity in corticothalamic system of cats attending to visual stimuli. *Neuroreport* 10 3589–3594. 10.1097/00001756-199911260-00023 10619649

[B6] Bekisz M. Wróbel A. (2003). Attention-dependent coupling between beta activities recorded in the cat’s thalamic and cortical representations of the central visual field. *Eur. J. Neurosci.* 17 421–426. 10.1046/j.1460-9568.2003.02454.x 12542680

[B7] Bell A. H. Malecek N. J. Morin E. L. Hadj-Bouziane F. Tootell R. B. Ungerleider L. G. (2011). Relationship between functional magnetic resonance imaging-identified regions and neuronal category selectivity. *J. Neurosci.* 31 12229–12240. 10.1523/JNEUROSCI.5865-10.2011 21865466 PMC3165163

[B8] Benjamini Y. Hochberg Y. (1995). Controlling the false discovery rate: a practical and powerful approach to multiple testing. *J. R. Stat. Soc. B Methodol.* 57 289–300.

[B9] Bethmann A. Tempelmann C. De Bleser R. Scheich H. Brechmann A. (2007). Determining language laterality by fMRI and dichotic listening. *Brain Res.* 1133 145–157. 10.1016/j.brainres.2006.11.057 17182011

[B10] Boksem M. A. Meijman T. F. Lorist M. M. (2005). Effects of mental fatigue on attention: an ERP study. *Brain Res. Cogn. Brain Res.* 25 107–116. 10.1016/j.cogbrainres.2005.04.011 15913965

[B11] Buffalo E. A. Fries P. Landman R. Liang H. Desimone R. (2010). A backward progression of attentional effects in the ventral stream. *Proc. Natl. Acad. Sci. U.S.A.* 107 361–365. 10.1073/pnas.0907658106 20007766 PMC2806732

[B12] Constantinidis C. Bucci D. J. Rugg M. D. (2013). Cognitive functions of the posterior parietal cortex. *Front. Integr. Neurosci.* 7 35 10.3389/978-2-88919-176-5 PMC3648698 23675328

[B13] Conway B. R. Moeller S. Tsao D. Y. (2007). Specialized color modules in macaque extrastriate cortex. *Neuron* 56 560–573. 10.1016/j.neuron.2007.10.008 17988638 PMC8162777

[B14] Crone N. E. Miglioretti D. L. Gordon B. Sieracki J. M. Wilson M. T. Uematsu S. (1998). Functional mapping of human sensorimotor cortex with electrocorticographic spectral analysis. I. Alpha and beta event-related desynchronization. *Brain* 121 2271–2299. 10.1093/brain/121.12.2271 9874480

[B15] de Haan E. H. Cowey A. (2011). On the usefulness of ’what’ and ’where’ pathways in vision. *Trends Cogn Sci.* 15 460–466. 10.1016/j.tics.2011.08.005 21906989

[B16] Debener S. Minow F. Emkes R. Gandras K. de Vos M. (2012). How about taking a low-cost, small, and wireless EEG for a walk? *Psychophysiology* 49 1617–1621. 10.1111/j.1469-8986.2012.01471.x 23013047

[B17] Dehaene S. Cohen L. (2011). The unique role of the visual word form area in reading. *Trends Cogn. Sci.* 15 254–262. 10.1016/j.tics.2011.04.003 21592844

[B18] Delorme A. Makeig S. (2004). EEGLAB: an open source toolbox for analysis of single-trial EEG dynamics. *J. Neurosci. Methods* 134 9–21. 10.1016/j.jneumeth.2003.10.009 15102499

[B19] Dikker S. Wan L. Davidesco I. Kaggen L. Oostrik M. McClintock J. (2017). Brain-to-brain synchrony tracks real-world dynamic group interactions in the classroom. *Curr. Biol.* 27 1375–1380. 10.1016/j.cub.2017.04.002 28457867

[B20] Dmochowski J. P. Sajda P. Dias J. Parra L. C. (2012). Correlated components of ongoing EEG point to emotionally laden attention – a possible marker of engagement? *Front. Hum. Neurosci.* 6:112 10.3389/fnhum.2012.00112 PMC3353265 22623915

[B21] Downing P. E. Chan A. W. Peelen M. V. Dodds C. M. Kanwisher N. (2006). Domain specificity in visual cortex. *Cereb. Cortex* 16 1453–1461. 10.1093/cercor/bhj086 16339084

[B22] Epstein R. Kanwisher N. (1998). A cortical representation of the local visual environment. *Nature* 392 598–601. 10.1038/33402 9560155

[B23] Faber L. G. Maurits N. M. Lorist M. M. (2012). Mental fatigue affects visual selective attention. *PLoS ONE* 7:e48073 10.1371/journal.pone.0048073 PMC3485293 23118927

[B24] Fukuda K. Dobbins J. G. Wilson L. J. Dunn R. A. Wilcox K. Woods D. S. (1997). An epidemiologic study of fatigue with relevance for the chronic fatigue syndrome. *J. Psychiatr. Res.* 31 19–29. 10.1016/S0022-3956(96)00046-5 9201644

[B25] Gandhi S. P. Heeger D. J. Boynton G. M. (1999). Spatial attention affects brain activity in human primary visual cortex. *Proc. Natl. Acad. Sci. U.S.A.* 96 3314–3319. 10.1073/pnas.96.6.3314 10077681 PMC15939

[B26] Gola M. Magnuski M. Szumska I. Wróbel A. (2013). EEG beta band activity is related to attention and attentional deficits in the visual performance of elderly subjects. *Int. J. Psychophysiol.* 89 334–341. 10.1016/j.ijpsycho.2013.05.007 23688673

[B27] Goodale M. A. Meenan J. P. Bülthoff H. H. Nicolle D. A. Murphy K. J. Racicot C. I. (1994). Separate neural pathways for the visual analysis of object shape in perception and prehension. *Curr. Biol.* 4 604–610. 10.1016/S0960-9822(00)00132-9 7953534

[B28] Habib R. Nyberg L. Tulving E. (2003). Hemispheric asymmetries of memory: the HERA model revisited. *Trends Cogn. Sci.* 7 241–245. 10.1016/S1364-6613(03)00110-4 12804689

[B29] Heathcote A. Popiel S. J. Mewhort D. J. (1991). Analysis of response time distributions: An example using the stroop task. *Psychol. Bull.* 109 340–347. 10.1037/0033-2909.109.2.340

[B30] Jason L. A. Evans M. Brown M. Porter N. (2010). What is fatigue? Pathological and nonpathological fatigue. *PM R* 2 327–331. 10.1016/j.pmrj.2010.03.028 20656613

[B31] Jung T.-P. Makeig S. Stensmo M. Sejnowski T. J. (1997). Estimating alertness from the EEG power spectrum. *IEEE Trans. Biomed. Eng.* 44 60–69. 10.1109/10.553713 9214784

[B32] Kant I. J. Bultmann U. Schroer K. Beurskens A. van Amelsvoort L. Swaen G. (2003). An epidemiological approach to study fatigue in the working population: the Maastricht cohort study. *Occup. Environ. Med.* 60 i32–i39. 10.1136/oem.60.suppl_1.i32 12782745 PMC1765733

[B33] Kasai K. Fukuda M. Yahata N. Morita K. Fujii N. (2015). The future of real-world neuroscience: imaging techniques to assess active brains in social environments. *Neurosci. Res.* 90 65–71. 10.1016/j.neures.2014.11.007 25433093

[B34] Klimesch W. (1999). EEG alpha and theta oscillations reflect cognitive and memory performance: a review and analysis. *Brain Res. Rev.* 29 169–195. 10.1016/S0165-0173(98)00056-3 10209231

[B35] Kravitz D. J. Saleem K. S. Baker C. I. Mishkin M. (2011). A new neural framework for visuospatial processing. *Nat. Rev. Neurosci.* 12 217–230. 10.1038/nrn3008 21415848 PMC3388718

[B36] Kravitz D. J. Saleem K. S. Baker C. I. Ungerleider L. G. Mishkin M. (2013). The ventral visual pathway: an expanded neural framework for the processing of object quality. *Trends Cogn. Sci.* 17 26–49. 10.1016/j.tics.2012.10.011 23265839 PMC3532569

[B37] Lilliefors H. (1967). On the Kolmogorov–Smirnov test for normality with mean and variance unknown. *J. Am. Stat. Associat.* 62 399–402. 10.1080/01621459.1967.10482916

[B38] Lin C.-T. Chuang C.-H. Wang Y.-K. Tsai S.-F. Chiu T.-C. Ko L.-W. (2012). Neurocognitive characteristics of the driver: a review on drowsiness, distraction, navigation, and motion sickness. *J. Neurosci. Neuroeng.* 1 61–81. 10.1166/jnsne.2012.1010

[B39] Lin C.-T. Huang K.-C. Chao C.-F. Chen J.-A. Chiu T.-W. Ko L.-W. (2010). Tonic and phasic EEG and behavioral changes induced by arousing feedback. *Neuroimage* 52 633–642. 10.1016/j.neuroimage.2010.04.250 20438854

[B40] Lin C.-T. Huang K.-C. Chuang C.-H. Ko L.-W. Jung T.-P. (2013). Can arousing feedback rectify lapses in driving? Prediction from EEG power spectra. *J. Neural. Eng.* 10:056024 10.1088/1741-2560/10/5/056024 24060726

[B41] Lin C.-T. Ko L.-W. Chung I.-F. Huang T.-Y. Chen Y.-C. Jung T.-P. (2006). Adaptive EEG-based alertness estimation system by using ICA-based fuzzy neural networks. *IEEE Trans. Circuits Syst. I* 53 2469–2476. 10.1109/TCSI.2006.884408

[B42] Macko K. A. Jarvis C. D. Kennedy C. Miyaoka M. Shinohara M. Sololoff L. (1982). Mapping the primate visual system with [2-14C] deoxyglucose. *Science* 218 394–397. 10.1126/science.7123241 7123241

[B43] Mackworth N. H. (1948). The breakdown of vigilance during prolonged visual search. *Q. J. Exp. Psychol.* 1 6–21. 10.1080/17470214808416738

[B44] Mahon B. Z. Milleville S. C. Negri G. A. Rumiati R. I. Caramazza A. Martin A. (2007). Action-related properties shape object representations in the ventral stream. *Neuron* 55 507–520. 10.1016/j.neuron.2007.07.011 17678861 PMC2000824

[B45] Makeig S. Jung T.-P. (1996). Tonic, phasic, and transient EEG correlates of auditory awareness in drowsiness. *Brain Res. Cogn. Brain Res.* 4 15–25. 10.1016/0926-6410(95)00042-9 8813409

[B46] Malach R. Reppas J. B. Benson R. R. Kwong K. K. Jiang H. Kennedy W. A. (1995). Object-related activity revealed by functional magnetic resonance imaging in human occipital cortex. *Proc. Natl. Acad. Sci. U.S.A.* 92 8135–8139. 10.1073/pnas.92.18.8135 7667258 PMC41110

[B47] Maunsell J. H. Treue S. (2006). Feature-based attention in visual cortex. *Trends Neurosci.* 29 317–322. 10.1016/j.tins.2006.04.001 16697058

[B48] McDowell K. Lin C.-T. Oie K. S. Jung T.-P. Gordon S. Whitaker K. W. (2013). Real-world neuroimaging technologies. *Access IEEE* 1 131–149. 10.1109/ACCESS.2013.2260791

[B49] McIntosh A. R. Grady C. L. Ungerleider L. G. Haxby J. V. Rapoport S. I. Horwitz B. (1994). Network analysis of cortical visual pathways mapped with PET. *J. Neurosci.* 14 655–666. 8301356 10.1523/JNEUROSCI.14-02-00655.1994 PMC6576802

[B50] Mishkin M. Ungerleider L. G. Macko K. A. (1983). Object vision and spatial vision: Two cortical pathways. *Trends Neurosci.* 6 414–417. 10.1016/0166-2236(83)90190-X

[B51] Murphey D. K. Yoshor D. Beauchamp M. S. (2008). Perception matches selectivity in the human anterior color center. *Curr. Biol.* 18 216–220. 10.1016/j.cub.2008.01.013 18258428

[B52] Ocklenburg S. Güntürkün O. (2012). Hemispheric asymmetries: the comparative view. *Front. Psychol.* 3:5 10.3389/fpsyg.2012.00005 PMC3266613 22303295

[B53] Osipova D. Takashima A. Oostenveld R. Fernández G. Maris E. Jensen O. (2006). Theta and gamma oscillations predict encoding and retrieval of declarative memory. *J. Neurosci.* 26 7523–7531. 10.1523/JNEUROSCI.1948-06.2006 16837600 PMC6674196

[B54] Pal N. R. Chuang C.-Y. Ko L.-W. Chao C.-F. Jung T.-P. Liang S.-F. (2008). EEG-based subject- and session-independent drowsiness detection: an unsupervised approach. *EURASIP J. Adv. Signal Process.* 2008:519480 10.1155/2008/519480

[B55] Palva S. Palva M. (2007). New vistas for alpha-frequency band oscillations. *Trends Neurosci.* 30 150–158. 10.1016/j.tins.2007.02.001 17307258

[B56] Poulsen A. T. Kamronn S. Dmochowski J. Parra L. C. Hansen L. K. (2017). EEG in the classroom: synchronised neural recordings during video presentation. *Sci. Rep.* 7:43916 10.1038/srep43916 PMC5339684 28266588

[B57] Rajkowski J. Kubiak P. Aston-Jones G. (1994). Locus coeruleus activity in monkey: phasic and tonic changes are associated with altered vigilance. *Brain Res. Bull.* 35 607–616. 10.1016/0361-9230(94)90175-9 7859118

[B58] Saproo S. Shih V. Jangraw D. C. Sajda P. (2016). Neural mechanisms underlying catastrophic failure in human–machine interaction during aerial navigation. *J. Neural Eng.* 13:066005 10.1088/1741-2560/13/6/066005 27705959

[B59] Sarter M. Givens B. Bruno J. P. (2001). The cognitive neuroscience of sustained attention: where top-down meets bottom-up. *Brain Res. Brain Res. Rev.* 35 146–160. 10.1016/S0165-0173(01)00044-3 11336780

[B60] Shomstein S. Behrmann M. (2006). Cortical systems mediating visual attention to both objects and spatial locations. *Proc. Natl. Acad. Sci. U.S.A.* 103 11387–11392. 10.1073/pnas.0601813103 16840559 PMC1544095

[B61] Tanaka M. Ishii A. Watanabe Y. (2015). Fatigue in the central nervous system. *Austin J. Clin. Neurol.* 2 1020.

[B62] Tsao D. Y. Moeller S. Freiwald W. A. (2008). Comparing face patch systems in macaques and humans. *Proc. Natl. Acad. Sci. U.S.A.* 105 19514–19519. 10.1073/pnas.0809662105 19033466 PMC2614792

[B63] Ungerleider L. G. Mishkin M. (1982). “Two cortical visual systems,” in *Analysis of Visual Behavior* eds Ingle D. J. Goodale M. A. Mansfield R. J. W. (Cambridge, MA: MIT Press) 549–586.

[B64] Vikingstad E. M. George K. P. Johnson A. F. Cao Y. (2000). Cortical language lateralization in right handed normal subjects using functional magnetic resonance imaging. *J. Neurol. Sci.* 175 17–27. 10.1016/S0022-510X(00)00269-0 10785252

[B65] Wróbel A. (2000). Beta activity: a carrier for visual attention. *Acta Neurobiol. Exp.* 60 247–260. 10.55782/ane-2000-1344 10909182

[B66] Yasui Y. (2009). A brainwave signal measurement and data processing technique for daily life applications. *J. Physiol. Anthropol.* 28 145–150. 10.2114/jpa2.28.145 19483376

[B67] Zander T. O. Kothe C. (2011). Towards passive brain-computer interfaces: applying brain-computer interface technology to human-machine systems in general. *J. Neural Eng.* 8:025005 10.1088/1741-2560/8/2/025005 21436512

